# Draft Genome Sequence of the Oleaginous Green Alga *Tetradesmus obliquus* UTEX 393

**DOI:** 10.1128/genomeA.01449-16

**Published:** 2017-01-19

**Authors:** B. M. Carreres, L. de Jaeger, J. Springer, M. J. Barbosa, G. Breuer, E. J. van den End, D. M. M. Kleinegris, I. Schäffers, E. J. H. Wolbert, H. Zhang, P. P. Lamers, R. B. Draaisma, V. A. P. Martins dos Santos, R. H. Wijffels, G. Eggink, P. J. Schaap, D. E. Martens

**Affiliations:** a Laboratory of Systems and Synthetic Biology, Wageningen University and Research, Wageningen, The Netherlands; b Bioprocess Engineering and AlgaePARC, Wageningen University and Research, Wageningen, The Netherlands; c Food and Biobased Research and AlgaePARC, Wageningen University and Research, Wageningen, The Netherlands; d Unilever Research and Development Vlaardingen, Vlaardingen, The Netherlands; e LifeGlimmer GmbH, Berlin, Germany; f Nord University, Bodø, Norway

## Abstract

The microalgae *Tetradesmus obliquus* is able to maintain a high photosynthetic efficiency under nitrogen limitation and is considered a promising green microalgae for sustainable production of diverse compounds, including biofuels. Here, we report the first draft whole-genome shotgun sequencing of *T. obliquus*. The final assembly comprises 108,715,903 bp with over 1,368 scaffolds.

## GENOME ANNOUNCEMENT

Microalgae are promising photosynthetic microorganisms for the sustainable production of many compounds ranging from bulk chemicals, such as biofuels, to high-value compounds such as pigments and polyunsaturated fatty acids (1). The freshwater microalga *Tetradesmus obliquus* (previously known as *Scenedesmus obliquus* and reclassified as *Acutodesmus obliquus* [2]) is a member of the *Chlorophyceae* and has four chromosomes (3). *T. obliquus* UTEX 393 was identified as a promising candidate for industrial applications, because it can accumulate triacylglycerol (TAG) up to 40% of its dry weight. Furthermore, *T. obliquus* is able to maintain a high photosynthetic efficiency under nitrogen limitation for a relatively long period, which results in a high yield of lipids on light and thus high volumetric and areal productivities (4–8).

The genome was sequenced using Illumina Hiseq2000, yielding nearly 200 million 101-bp reads, with an estimated average insert size of 248 bp. Additionally, more than 8 million 51-bp mate pairs were sequenced, with an estimated average insert size of 4,139 bp. Two methods were used in parallel for assembling and scaffolding, and the resulting scaffold sets were merged. In the first method, contigs were assembled using IDBA-UD version 1.1.1 (9), producing a 96,197,736-bp assembly in 9,191 contigs, and scaffolded using SOPRA version 1.4.6 (10), as previously recommended (11), to obtain 2,509 scaffolds. In the second method, contigs were assembled using CLC Genomics Workbench version 5.1, producing a 93,347,907-bp assembly in 10,609 contigs, and scaffolded using SSPACE to obtain 2,768 scaffolds (12). The two scaffold sets were merged using *quickmerge* (13) with suitable settings. Additionally, the scaffolds matching 96% of their length over another scaffold were taken out. Between each scaffolding or merging iteration, the gaps were closed using GapFiller (14). The result was 1,368 scaffolds with a total length of 107,715,903 bp. Using QUAST version 4.3 (15), we compared our assembly to the complete chloroplast genome sequence of UTEX-393 (16), covering 99.97% (~50-bp gap) of the reference with few overlapping scaffolds.

### Accession number(s).

This whole-genome shotgun project has been deposited at DDBJ/ENA/GenBank under the accession numbers FNXT01000001 to FNXT01001368. The versions described are the first versions, FNXT01000001 to FNXT01001368.
